# Bee Venom Suppresses the Differentiation of Preadipocytes and High Fat Diet-Induced Obesity by Inhibiting Adipogenesis

**DOI:** 10.3390/toxins10010009

**Published:** 2017-12-24

**Authors:** Se-Yun Cheon, Kyung-Sook Chung, Seong-Soo Roh, Yun-Yeop Cha, Hyo-Jin An

**Affiliations:** 1 Department of Pharmacology, College of Korean Medicine, Sang-ji University, Wonju-si, Gangwon-do 26339, Korea; chunsay1008@naver.com; 2 Catholic Precision Medicine Research Center, College of Medicine, The Catholic University of Korea, 222, Banpo-daero, Seocho-gu, Seoul 06591, Korea; adella76@hanmail.net; 3 Department of Herbology, College of Korean Medicine, Daegu Hanny University, Suseong-gu, Deagu 42158, Korea; ddede@dhu.ac.kr; 4 Department of Rehabilitation Medicine of Korean Medicine and Neuropsychiatry, College of Korean Medicine, Sang-ji University, Wonju-si, Gangwon-do 26339, Korea; omdcha@sangji.ac.kr

**Keywords:** Bee venom, PPARγ, AMPK, MAPK, adipogenesis

## Abstract

Bee venom (BV) has been widely used in the treatment of certain immune-related diseases. It has been used for pain relief and in the treatment of chronic inflammatory diseases. Despite its extensive use, there is little documented evidence to demonstrate its medicinal utility against obesity. In this study, we demonstrated the inhibitory effects of BV on adipocyte differentiation in 3T3-L1 cells and on a high fat diet (HFD)-induced obesity mouse model through the inhibition of adipogenesis. BV inhibited lipid accumulation, visualized by Oil Red O staining, without cytotoxicity in the 3T3-L1 cells. Male C57BL/6 mice were fed either a HFD or a control diet for 8 weeks, and BV (0.1 mg/kg or 1 mg/kg) or saline was injected during the last 4 weeks. BV-treated mice showed a reduced body weight gain. BV was shown to inhibit adipogenesis by downregulating the expression of the transcription factors CCAAT/enhancer-binding proteins (C/EBPs) and the peroxisome proliferator-activated receptor gamma (PPARγ), using RT-qPCR and Western blotting. BV induced the phosphorylation of AMP-activated kinase (AMPK) and acetyl-CoA carboxylase (ACC) in the cell line and in obese mice. These findings demonstrate that BV mediates anti-obesity/differentiation effects by suppressing obesity-related transcription factors.

## 1. Introduction

Obesity is defined as an abnormal or excessive fat accumulation that presents a risk to health. Adipocyte hyperplasia and hypertrophy are determinant factors of obesity [1]. Adipocytes differentiate from stem cells or other precursor cells [2]. Differentiating and maturing adipocytes involve a complex program of gene expression that is important for obesity-related diseases [3]. 3T3-L1 preadipocytes have been used in studies regarding adipogenesis and differentiation. These cells differentiate in response to adipogenic inducers including insulin, dexamethasone, and 3-isobutyl-1-methylxanthine (IBMX) [4]. The differentiation sequence from preadipocytes to adipocytes comprises confluence, mitotic clonal expansion (MCE), and terminal differentiation. In the first stage, confluent cells enter a growth arrest phase [5,6]. These growth-arrested cells subsequently restart the cell cycle and increase cell numbers three- to four-fold during the MCE phase [7]. This hyperplasia during cell differentiation is related to the production of specific adipogenic transcription factors [8].

Peroxisome proliferator-activated receptors (PPAR)γ and CCAAT/enhancer-binding proteins (C/EBPs) promote the differentiation of adipocytes [9]. During early-stage differentiation of 3T3-L1 cells, the expression of C/EBPβ and C/EBPδ is increased after hormonal induction, followed by increases in the expression of C/EBPα and PPARγ [10]. C/EBPδ is important for MCE to occur during differentiation in the early stage of adipogenesis [11]. Gene expression of C/EBPβ induces the expression of PPARγ and C/EBPα [12,13]. The activation of the C/EBP family and of PPARγ regulates the expression of various adipogenic factors that promote fat accumulation.

Adenosine monophosphate–activated protein kinase (AMPK), known as a regulator of energy homeostasis, is an important target for controlling obesity [14]. In adipogenesis, AMPK activation regulates glucose and lipid metabolism by inactivating metabolic enzymes [15]. Phosphorylation by AMPK inactivates acetyl-coenzyme A carboxylase (ACC) and 3-hydroxy-3-methylglutaryl-coenzyme A reductase (HMGCR), leading to the inhibition of fatty acids and cholesterol syntheses, as well as increased fatty acid oxidation [16]. AMPK regulates the channeling of acyl-CoA towards β-oxidation and lipid biosynthesis, leading to the inhibition of glycerol-3-phosphate acyltransferase (GPAT) [17]. In addition, the phosphorylation of AMPK also inhibits the expression of adipogenic transcription factors, such as C/EBPβ, C/EBPδ, C/EBPα, and PPARγ [18].

Bee venom (BV), a complex mixture of proteins, peptides, and low molecular weight components, is an effective defense tool used for the protection of the hive by the honey bee [19]. Despite causing pain to humans who are stung, BV has been used as a traditional medicine to treat a diverse range of conditions, including tumors, skin diseases, and pain [20]. It has also been reported that the inhibition of atherosclerotic lesions via anti-inflammatory mechanisms and the suppression of benign prostatic hyperplasia in rats are additional beneficial properties [21,22]. BV is known to contain a complex mixture of active enzymes and peptides, including phospholipase A2, melittin, and apamin [23]. Melittin is a major component of BV that has been shown to improve atherosclerotic lesions and to downregulate pro-inflammatory cytokines, adhesion molecules, proatherogenic proteins, and the NF-κB signal pathway in high-fat treated atherosclerotic animal models [24]. In addition, apamin attenuated lipids, proinflammatory cytokines, adhesion molecules, fibrotic factors, and macrophage infiltration in LPS/fat-induced atherosclerotic mice [25]. BV was also reported to exhibit anti-obesity effects [26] but the mechanism has not yet been clarified. In the present study, we investigated the anti-obesity effects of BV in 3T3-L1 preadipocytes and in an HFD-induced obesity animal model.

## 2. Results

### 2.1. BV Suppressed Cell Hyperplasia and Lipid Accumulation during Differentiation of 3T3-L1 Adipocytes

**Figure 1:**
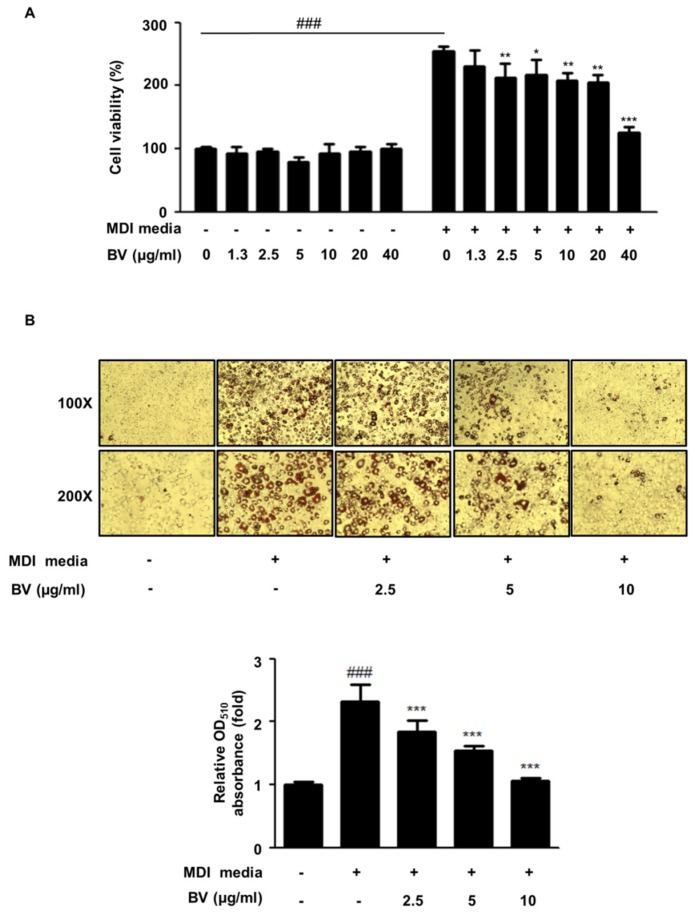
Bee venom (BV) suppressed lipid accumulation in 3T3-L1 preadipocytes. (**A**) Cells were cultured in the growth medium or the differentiation medium containing concentrations ranging from 1.3 to 40 μg/mL of BV for 3 days. (**B**) Preadipocytes were differentiated with and without BV (2.5, 5, and 10 μg/mL) for 9 days. Differentiated cells were stained with Oil red O and images were taken with a Leica DM IL LED microscope (100× and 200× magnifications). (**C**) Oil red O was extracted from lipid droplets using isopropanol and was measured at 510 nm. ^###^ *p* < 0.001 vs. non-differentiation cells. * *p* < 0.05, ** *p* < 0.01, *** *p* < 0.001 vs. differentiation cells.

Hyperplasia and lipid accumulation are known to occur in the 3T3-L1 cell line during differentiation [8,27]. 3T3-L1 cells were treated with BV (concentrations from 1.25 to 40 μg/mL) in the differentiation media (MDI) or the growth media (BS). After treatment with BV, the cell viability was determined by a 3-(4,5-Dimethylthiazol-2-yl)-2,5-diphenyl tetrazolium bromide (MTT) assay (Figure 1A). BV had no effect on the cell viability in the culture media. However, BV significantly reduced the cell viability at 2.5 μg/mL or higher concentrations during the differentiation of adipocytes.

To determine the inhibitory effect of BV on lipid accumulation in adipocytes, 3T3-L1 cells were treated with and without BV (2.5, 5, or 10 μg/mL) for 9 days. As shown in Figure 1B, differentiation-induced adipocytes drastically increased their lipid storage approximately 2.32-fold, compared with undifferentiated cells. In contrast, treatment with BV significantly reduced lipid droplet accumulation in a dose-dependent manner.

### 2.2. BV Suppressed the Expression of Adipogenic Markers during Differentiation of 3T3-L1 Adipocytes

**Figure 2:**
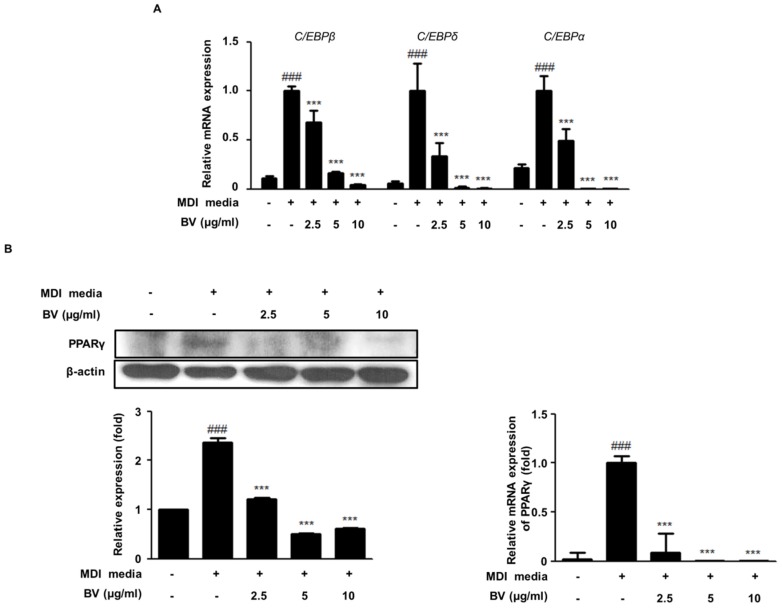
The effects of BV on the expression levels of lipid metabolism and adipogenesis in 3T3-L1 cells. (**A**) The mRNA levels of C/EBPβ, C/EBPδ, and C/EBPα were measured by qRT-PCR. (**B**) The protein and the mRNA expression levels of PPARγ were determined by Western blot analysis or by qRT-PCR. β-actin was used as an internal control. ^###^ *p* < 0.001 vs. non-differentiation cells, *** *p* < 0.001 vs. differentiation cells.

Transcription factors, such as PPARγ and the C/EBP family, are known to play important roles during the maturation and the differentiation of adipocytes [2,28]. In this study, we investigated the anti-adipogenesis activity of BV in differentiated adipocytes using qRT-PCR and Western blot analysis. As shown in Figure 2, the mRNA expression of C/EBPα, C/EBPβ, C/EBPδ, and PPARγ was upregulated in differentiation-induced adipocytes, compared to what was seen in undifferentiated cells. However, the treatment with BV significantly downregulated the mRNA expression of the C/EBP family and decreased PPARγ expression (both mRNA and protein levels; Figure 2B).

### 2.3. BV Regulated the MAPK Pathway during Differentiation of 3T3-L1 Preadipocytes

**Figure 3:**
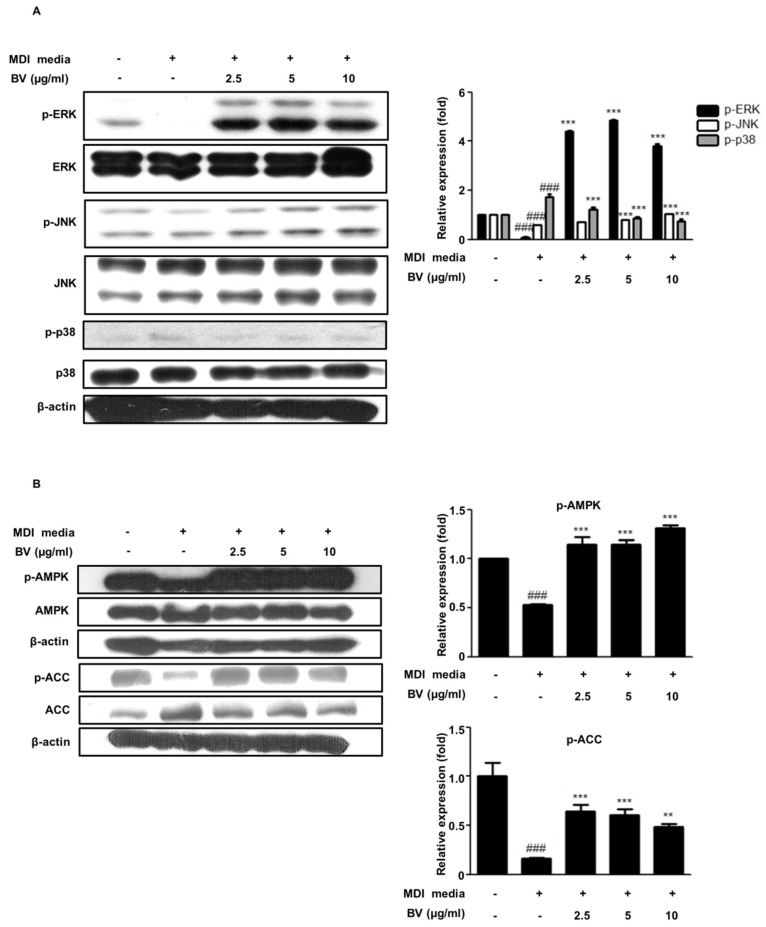
The effects of BV on the mitogen-activated protein kinase (MAPK) and the adenosine monophosphate-activated protein kinase (AMPK) pathways in 3T3-L1 preadipocytes. 3T3-L1 cells were treated with BV (2.5, 5, and 10 μg/mL for 3 days). The total cell lysates were analyzed by Western blotting to determine the expression of (**A**) MAPKs (**B**) AMPK and acetyl coenzyme A carboxylase (ACC). β-actin was used as an internal control. ^###^ *p* < 0.001 vs. non-differentiation cells. ** *p* < 0.01, *** *p* < 0.001 vs. differentiation cells.

Mitogen-activated protein kinases (MAPKs) play a crucial role in many essential cellular responses, including proliferation, apoptosis, and differentiation [29]. In particular, MAPKs are associated with regulatory effects on adipocyte differentiation [30]. To investigate the effect of BV on the MAPK pathway, we determined the protein expression of factors involved in the MAPK pathway using Western blot analysis in differentiated adipocytes. As shown in Figure 3A, the phosphorylation of ERK and JNK decreased, whereas the phosphorylation of p38 increased during differentiation, compared to that of the undifferentiated control cells. The phosphorylation of ERK and JNK was upregulated in the BV-treated adipocytes and the phosphorylation of p38 was significantly decreased.

### 2.4. BV Activated the AMPK Pathway during the Differentiation of 3T3-L1 Preadipocytes

AMPK, a key enzyme of energy metabolism, regulates glucose and lipid metabolism [31]. The phosphorylation of AMPK inhibits the expression of adipogenic transcription factors, such as C/EBPs and PPARγ [18]. To investigate the effect of BV on the activation of AMPK, we determined the degree of AMPK phosphorylation using Western blotting. As shown in Figure 3B, AMPK phosphorylation was decreased in the differentiated 3T3-L1 preadipocytes, compared with the undifferentiated control cells. Meanwhile, our results revealed that treatment with BV significantly recovered the AMPK phosphorylation in differentiated adipocytes.

### 2.5. BV Suppressed Body Weight, Fat, and Lipid Accumulation in HFD-Induced Obese Mice

**Figure 4:**
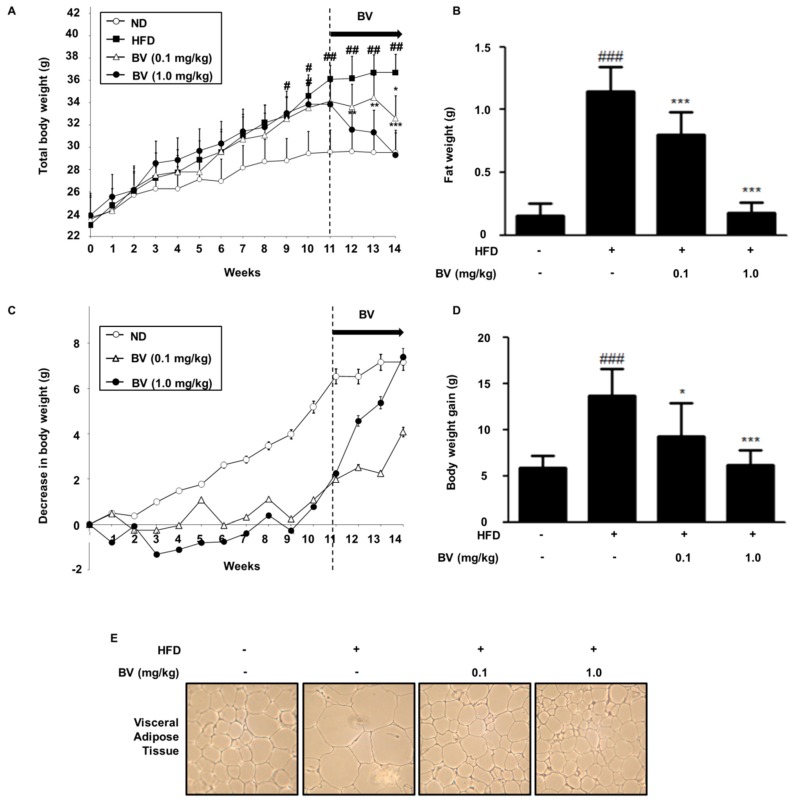
Effects of BV injection on (**A**) total body weight, (**B**) fat weight, (**C**) a decrease in body weight, (**D**) body weight gain, and (**E**) Epididymis adipose tissue (100× magnifications) from mice. The duration of the experimental window was 14 weeks during which mice were fed on normal diet or a high-fat diet. The representative photographic images of mice were from different treatment/feeding groups at the time of sacrifice. Values are expressed as the mean ± SEM of 10 mice per group. ^#^ *p* < 0.05, ^##^ *p* < 0.01, ^###^ *p* < 0.001 vs. the normal diet (ND) group. * *p* < 0.05, ** *p* < 0.01, *** *p* < 0.001 vs. the high fat diet (HFD) group.

As shown in Figure 4, the total body weight of mice in the high fat diet (HFD) group, including both fat and body weight gain, were significantly increased, compared to that of the normal diet (ND) group. In contrast to the HFD group, the BV injection suppressed the total body weight, fat, and body weight gain. Obesity is characterized by the hypertrophy and the hyperplasia of adipose tissue [32], therefore we examined the inhibitory effect of BV using hematoxylin and eosin (H&E) staining. As shown in Figure 4E, H&E analysis data indicated that the HFD-fed mice showed hypertrophy of adipocytes in the epididymal adipose tissue, whereas BV treated groups showed an inhibited hypertrophy of adipocytes.

### 2.6. BV Suppressed Adipogenic Markers and Activated the AMPK Pathway in HFD-Induced Obese Mice

The adipogenic transcription factors, PPARγ and C/EBPα, regulate the genes for fat accumulation. Several in vivo studies have shown that PPARγ plays a crucial role in adipogenesis [9,33]. The inhibition of adipogenesis in the BV-treated groups was investigated and was found to be associated with molecular signaling, by the adipogenic markers involved in lipid metabolism and adipogenesis. We examined the protein expression of PPARγ and C/EBPα in adipose tissue, and it was significantly upregulated in the HFD group. In contrast, the expression of factors related to adipogenesis decreased in the BV-treated groups, compared to the expression in the HFD group.

**Figure 5:**
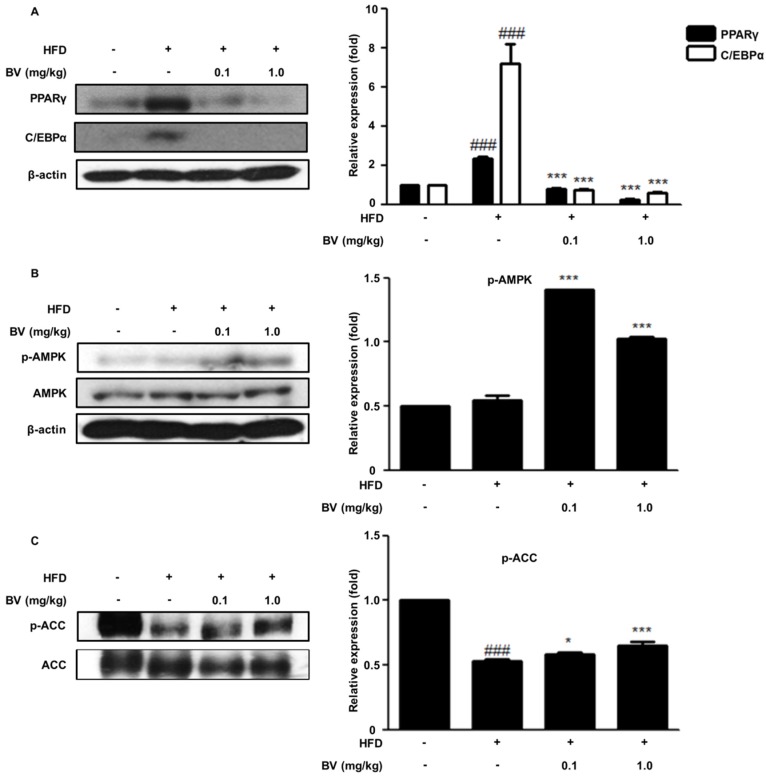
BV regulated adipogenic markers and the AMPK pathway in HFD-induced mice. (**A**) Important adipogenic transcription factors, PPAR γ and C/EBPα, determined using Western blot analysis. (**B**) AMPK and (**C**) ACC determined using Western blot analysis. β-actin was used as an internal control. ^###^ *p* < 0.001 vs. ND group. * *p* < 0.05, *** *p* < 0.001 vs. HFD group.

The activation of AMPK to p-AMPK inhibited the activity of ACC, which resulted in an increase of carnitine palmitoyl-CoA transferase-1 [16]. ACC regulates the metabolism of fatty acids, and in particular, the ACC1 isoform of ACC regulates fatty acid synthesis [34]. In experiments with mice, the phosphorylated level of AMPK was not significantly altered in the HFD-fed group. However, the phosphorylation of ACC in mice was downregulated by HFD. BV treatment significantly increased the phosphorylated levels of AMPK and of ACC (Figure 5B,C).

## 3. Discussion

Adipocyte differentiation and lipid accumulation are both processes related to the development of obesity [35]. The 3T3-L1 cell line was derived from Swiss 3T3 mouse embryos that are used as a model of adipocyte differentiation. Adipocyte differentiation is regulated by signaling molecules from numerous pathways [4]. In murine preadipocyte models, differentiation proceeds as follows: achievement of one hundredth confluence and growth arrest, hormonal induction, re-entry into the cell cycle, post-confluent mitosis, known as MCE. MCE is an important step in the differentiation of adipocytes [8]. During MCE, the number of cells increases 3- to 4-fold [7]. Several studies have identified that the suppression of adipocyte differentiation occurs through the inhibition of MCE [36,37]. Therefore, the differentiation of preadipocytes and their proliferation are two intimately linked processes.

We investigated the inhibitory effect of BV on adipocyte proliferation by determining the effect of BV on MCE in differentiating preadipocytes, by applying the MTT assay. As shown in Figure 1, differentiating cells markedly increased cell numbers, compared to the preadipocyte with BS media (DMEM + 10% BS media). BV treatment inhibited cell proliferation in a dose-dependent manner. BV showed inhibitory effects at the lowest investigated concentration of 2.5 μg/mL. In addition, lipid accumulation was determined by Oil Red O staining (Figure 1B) on Day 8. The data indicated that BV suppressed the MCE process in MDI-induced 3T3-L1 preadipocytes. Our results demonstrate that BV significantly reduced lipid accumulation in differentiating 3T3-L1 cells.

As differentiation progresses, lipid accumulation and numerous adipogenic genes upregulate adipogenesis through the adipocyte-specific transcription factors, C/EBPs and PPARγ [38,39]. C/EBPβ and C/EBPδ, the first transcription factors in directing the differentiation process, are increased after induction of differentiation. C/EBPβ is responsive mainly to DEX and C/EBPδ is responsive mainly to IBMX. After removal of these differentiation inducers, expression of C/EBPβ and C/EBPδ are decreased. In addition, C/EBPβ and δ are known to mediate the expression of PPARγ and C/EBPα [10,40]. C/EBPδ is related to the expression of C/EBPβ in the early phase of adipogenesis [11]. In this study, BV decreased the expression of C/EBPs and PPARγ in 3T3-L1 preadipocytes (Figure 2) and in adipose tissue in the HFD-fed obese mice (Figure 5A). Our findings suggest that BV inhibits early adipogenic processes and lipid accumulation by downregulating the expression of C/EBPs and PPARγ.

In this study, we induced obesity by feeding a high-fat diet to mice for 11 weeks, followed by an injection of BV for 4 weeks. The BV injected group exhibited decreased HFD-induced body and fat weight (Figure 4). Increase of body and fat weight are closely related warning signs for health issues [41], i.e., the accumulation of epididymis adipose tissue is related to metabolic problems, such as insulin resistance, hypertension, and elevated plasma triglyceride levels [42,43]. Histological analysis revealed a greater number of hypertrophied cells in the adipose tissue of the HFD group, whereas the BV injection suppressed adipocyte size in HFD-induced adipose tissue (Figure 4). This result indicates that BV inhibits the hypertrophy of adipocytes in HFD-fed obese mice.

The activity of AMPK in adipose tissue is a useful marker for metabolic disease [44]. ACC, which is a major fatty acid synthetic enzyme, is reduced by the activation of AMPK [45]. It is well known that aminoimidazole-4-carboxamide riboside (AICAR) and metformin, known as AMPK activators, decrease the transcriptional activity of the PPARγ/retinoid X receptor (RXR) in the rat hepatoma cell line H4IIEC3, whereas compound C (6-4[4-(2-piperidin-1-yl-ethoxy)-phenyl]-3-pyridin-4-yl-pyrazolo[1,5-*a*]pyrimidine), known as an AMPK inhibitor, reversed the effects of AICAR and metformin [46]. Furthermore, it has been reported that metformin decreased the plasma levels of glucose and triglycerides by inhibiting sterol regulatory element-binding protein (SREBP)-1 activity [47]. Our present results indicated that BV enhanced the phosphorylation of AMPK and ACC during the differentiation of cultured adipocytes and in the HFD-induced obese mice (Figure 3B and Figure 5B,C). These data suggest that BV regulates the AMPK pathway, which may be involved in the fatty acid metabolism in HFD-fed obese mice.

MAPKs are key regulators of cell growth factors, cytokines, cell proliferation, differentiation, motility, and many other cellular processes [48,49]. The MAPKs are divided into three main groups: the ERK 1/2, the c-Jun NH2-terminal kinases (JNK 1/2/3), and the p38 MAP kinases [50]. ERK 1/2 is involved in the differentiation of adipocytes; however, continual activation inhibits the differentiation of adipocytes [30,51]. Downregulation of ERK1/2 led to a reduction in adipocyte differentiation [52]. However, some studies reported that ERK activation attenuates the differentiation of adipocytes [53,54]. JNK is known to be involved in insulin resistance [55]. However, a previous study reported that the inhibition of JNK increased lipid accumulation and the expression of PPARγ in 3T3-L1 adipocytes [56]. P38 MAPK plays a key role in adipogenesis. SB203580, an inhibitor of p38 MAP kinase, blocks adipogenesis during only the early stages of adipocyte differentiation [57]. Our results showed that the BV treatment of cells enhanced the phosphorylation of ERK and JNK (Figure 3A). However, BV did not change the p38 phosphorylation. These data suggest that BV suppressed lipid accumulation and regulated adipogenic factors by regulating MAPK signaling.

In conclusion, the present study has demonstrated that BV inhibits early adipogenic processes by downregulating the MCE stage by regulating C/EBPs, PPARγ, ERK, and AMPK signaling. Based on these findings, we conclude that BV may be a useful preventive and therapeutic agent in the treatment of obesity.

## 4. Materials and Methods

### 4.1. Chemicals and Reagents

BV, IBMX, Dexamethasone (DEX), insulin, Oil red O, and all other chemicals were purchased from Sigma Chemical Co. (St. Louis, MO, USA). Dulbecco’s modified Eagles medium (DMEM), bovine serum (BS), fetal bovine serum (FBS), and penicillin-streptomycin (PS) were purchased from Life Technologies, Inc. (Grand Island, NY, USA). Antibodies against PPARγ (E-8; cat. no. sc-7273), C/EBPα (C-18; cat. no. sc-9314), and β-actin (C4; cat. no. sc-47778) were purchased from Santa Cruz biotechnology, Inc. (Santa Cruz, CA, USA). Phospho-extracellular signal-regulated kinase (p-ERK; Thr202/Tyr204; cat. no. #9101), ERK (cat. no. #9102), phospho-stress-activated protein kinase/Jun-amino-terminal kinase (p-JNK; Thr183/Tyr185; cat. no. #9251), JNK (cat. no. #9252), phospho-p38 MAPK (p-p38; Thr180/Thy182; cat. no. #9215), p38 (cat. no. #9212), p-AMPK (Thr172; cat. no. #2535), AMPK (cat. no. #2532), p-ACC (Ser79; cat. no. #3661), and ACC (cat. no. #3662) antibodies were purchased from Cell Signaling Technology, Inc. (Danvers, MA, USA) Horseradish peroxidase conjugated secondary antibodies were purchased from Jackson ImmunoResearch Laboratories, Inc. (West Grove, PA, USA). SYBR Green Master Mix was purchased from Applied Biosystems (Foster, CA, USA). *C/EBPα*, *C/EBPβ, C/EBPδ, PPARγ*, and glyceraldehyde-3-phosphate dehydrogenase (*GAPDH*) oligonucleotide primers were purchased from Bioneer (Daejeon, Korea).

### 4.2. Cell Culture and Treatment

Preadipocytes, 3T3-L1, were purchased from the Korean Cell Line Bank (Seoul, Korea) and were cultured in DMEM, supplemented with 10% BS, penicillin (100 U/mL), and 100 μg/mL streptomycin in an incubator at 37 °C with 5% CO_2_. The analysis of adipocyte differentiation was carried out by culturing 3T3-L1 cells in 60 mm dishes at a density of 2 × 10^5^ cells per mL to confluence. At full confluence, the cells were first differentiated with MDI media (0.5 mM IBMX, 1 μg/mL insulin, and 1 μM DEX in DMEM containing 10% (*v*/*v*) FBS and 1% PS). During this stage, we treated plates with various concentrations of BV. During the second stage, commencing on Day 3 of differentiation, the cells were treated with 1 μg/mL insulin in DMEM with 10% (*v*/*v*) FBS and 1% PS. In the final stage, cells were transferred to DMEM with 10% FBS and 1% PS, and the culture media was changed every 3 days. The differentiation of 3T3-L1 required 3 days in each stage.

### 4.3. MTT Assay

Cell viability was assessed using the 3-(4,5-Dimethylthiazol-2-yl)-2,5-diphenyl tetrazolium bromide (MTT) assay. Briefly, the 3T3-L1 preadipocyte cells were seeded into a 96-well plate at a density of 1 × 10^4^ cells per well and were treated with various concentrations (1.25 to 40 μg/mL) of BV for 72 h at 37 °C in humidified air with 5% CO_2_. After the treatment, the cells were stained by adding MTT solution (5 mg/mL) for 4 h at 37 °C. After removing the excess reagent, the insoluble formazan product was dissolved in DMSO. The cell viability was measured at 570 nm using an Epoch^®^ microvolume spectrophotometer (BioTek Instruments Inc., Winooski, VT, USA).

### 4.4. Oil Red O Staining

In order to observe lipid accumulation in the 3T3-L1 adipocytes, the differentiated adipocytes were stained with Oil Red O. As described above, the differentiation was initiated by exchanging the medium and by adding BV at three concentrations. Following the differentiation, 3T3-L1 adipocytes were washed three times with phosphate-buffered saline (PBS, pH = 7.4) and were fixed with 10% formaldehyde solution in PBS for 1 h at 25 °C. After washing with distilled water three times, the cells were then stained with 3 mg/mL Oil Red O dye solution in 60% isopropanol for 2 h at room temperature. Any excess Oil Red O dye was washed away with distilled water. The images of the Oil Red O-stained adipocytes were acquired using a Leica DM IL LED microscope (Leica, Wetzlar, Germany). The intracellular lipid content was measured by extracting Oil Red O with isopropanol, and the absorbance at 520 nm was recorded using an Epoch^®^ microvolume spectrophotometer (BioTek Instrument, Inc., Winooski, VT, USA).

### 4.5. Western Blot Analysis

The cells were lysed, and the tissue was homogenized in PRO-PREP™ protein extraction solution (Intron Biotechnology, Seoul, Korea) and was then incubated for 20 min at 4 °C. Debris was removed by microcentrifugation at 11,000× *g*, followed by a quick freezing of the supernatants. The protein concentration was determined using the Bio-Rad protein assay reagent according to the manufacturer’s instructions (Bio-Rad, Hercules, CA, USA). Proteins were electro-blotted onto a polyvinylidene difluoride (PVDF) membrane following their separation on an 8–12% SDS polyacrylamide gel. The membrane was incubated for 1 h with a blocking solution (5% skim milk) at room temperature, followed by incubation with a 1:1,000 dilution of primary antibodies, including, PPARγ, C/EBPα, p-ERK, ERK, p-JNK, JNK, p-p38, p38, p-AMPK, AMPK, p-ACC, ACC, and β-actin, overnight at 4 °C. The blots were washed three times with Tween 20/Tris-buffered saline (T/TBS) and were then incubated in a horseradish peroxidase-conjugated secondary antibody (dilution, 1:2500) for 2 h at room temperature. After washing them three times in T/TBS, the immuno-detection bands were reacted with the ECL solution (Ab signal, Seoul, Korea) and were recorded on X-ray film (Agfa, Belgium).

### 4.6. Isolation of Total RNA and Reverse Transcription Quantitative Polymerase Chain Reaction (RT-qPCR)

The cells were homogenized, and the total RNA was isolated using a Trizol reagent (Invitrogen, Carlsbad, CA, USA). cDNA was obtained using the isolated total RNA (1 μg), a d(T)16 primer, and avian myeloblastosis virus reverse transcriptase (AMV-RT). The relative gene expression was quantified using real-time PCR (Real-Time PCR System 7500, Applied Biosystems, Foster city, CA, USA) with a SYBR green PCR master mix (Applied Biosystems, Foster city, CA, USA). The forward and reverse primers were as follows: *PPARγ*, 5′-ATCGAGTGCCGAGTCTGTGG-3′ and 5′-GCAAGGCACTTCTGAAACCG-3′; *C/EBPα*, 5′-GGAACTTGAAGCACAATCGATC-3′ and 5′-TGGTTTAGCATAGACGTGCACA-3′; *C/EBPβ*, 5′-GGGGTTGTTGATGTTTTTGG-3′ and 5′-CGAAACGGAAAAGGTTCTCA-3′; *C/EBPδ*, 5′-GATCTGCACGGCCTGTTGTA-3′ and 5′-CTCCACTGCCCACCTGTCA-3′; *GAPDH*, 5′-GACGGCCGCATCTTCTTGT-3′ and 5′-CACACCGACCTTCACCATTTT-3′.

The gene Ct values of *PPARγ*, *C/EBPα*, *C/EBPβ*, and *C/EBPδ* were normalized using the Gene Express 2.0 program (Applied Biosystems, Foster city, CA, USA) to the Ct value of *GAPDH*.

### 4.7. Animals

C57BL/6 mice (6 weeks old, male) were purchased from Daehan Biolink Co. Ltd. (Daejeon, Korea) and were maintained under constant conditions (temperature, 22 ± 3 °C; humidity, 40–50%; light/dark cycle 12/12 h). The mice were adapted to the feeding conditions for 1 week and then were provided free access to food and tap water for 14 weeks. The mice were randomly separated into groups of four each: ND (normal diet), HFD (high-fat diet, 30% fat) only, and the BV-treated groups (0.1 or 1.0 mg/kg i.p.; high-fat diet). Their body weight and dietary intake were recorded every week. On the last day of the 14th week, the animals were fasted overnight. Blood samples were collected for lipid profiling, and the adipose tissue was excised, rinsed, and stored at −80 °C until analysis. The Institutional Animal Care and Use Committee (IACUC) of the College of Sang-ji University of Korea approved the study protocol. The approval code is 2014-10 and the approval date is 22 July 2014.

### 4.8. Histological Analysis

The adipose samples were fixed in 10% formalin and were embedded in paraffin; the sections were of 8-μm thickness. The sections were stained with hematoxylin and eosin (H&E) for the histological analysis of fat droplets. Images were acquired using a Leica DM IL LED microscope (Leica, Wetzlar, Germany).

### 4.9. Analysis of Serum Lipid Profiles

The blood samples were collected and centrifuged at 1003× *g*, for 15 min at room temperature to obtain serum samples, which were immediately frozen at −80 °C for further measurements. The serum concentrations of triglyceride and LDL cholesterol were determined by enzymatic methods with commercial kits (BioVision; Milpitas, CA, USA).

### 4.10. Statistical Analysis

The data are expressed as mean ± standard deviation (SD) of triplicate experiments. Statistical significance was determined using ANOVA and Dunnett’s post hoc test, and *p*-values of less than 0.05 were considered statistically significant.
